# Chest pain presentations to hospital during the COVID-19 lockdown: Lessons for public health media campaigns

**DOI:** 10.1371/journal.pone.0249389

**Published:** 2021-04-01

**Authors:** Amy V. Ferry, Collette Keanie, Martin A. Denvir, Nicholas L. Mills, Fiona E. Strachan

**Affiliations:** 1 BHF Centre for Cardiovascular Science, University of Edinburgh, Edinburgh, United Kingdom; 2 Department of Cardiology, Royal Infirmary of Edinburgh, Edinburgh, United Kingdom; 3 Usher Institute of Population Health Sciences and Informatics, University of Edinburgh, Edinburgh, United Kingdom; University of Birmingham, UNITED KINGDOM

## Abstract

**Objective:**

Emergency Department (ED) attendances with chest pain reduced during the COVID-19 lockdown. We performed a service evaluation project in NHS Lothian to explore how and why the COVID-19 pandemic and public health advice had affected chest pain presentations and help-seeking behaviour at an individual patient level using a qualitative interview approach.

**Methods:**

We carried out 28 semi-structured telephone interviews with a convenience sample of patients who presented with chest pain during lockdown and in patients with known coronary heart disease under the outpatient care of a cardiologist in April and May 2020. Interviews were audio recorded and voice files listened to while making detailed notes. Salient themes and issues were documented as verbatim extracts. Interviews were analysed thematically.

**Results:**

Patient interviews revealed three main themes. 1) pandemic help-seeking behaviour; describing how participants made the decision to seek professional healthcare assessment. 2) COVID-19 exposure concerns; describing how the subthemes of perceived vulnerability, wishing to protect others and adding pressure to the health service shaped their decision making for an episode of acute chest pain. 3) hospital experience; describing the difference between the imagined and actual experience in hospital.

**Conclusions:**

Qualitative interviews revealed how the pandemic shaped help-seeking practices, how patients interpreted their personal vulnerability to the virus, and described patient experience of attending hospital for assessment during this time. As patient numbers presenting to hospital appeared to mirror public health messaging, dynamic monitoring of this messaging should evaluate public response to healthcare campaigns to ensure the net impact on health, pandemic and non-pandemic related, is optimised.

## Introduction

Symptoms suggestive of acute coronary syndrome are one of the most common reasons for Emergency Department presentation [1]. Reports from around the world have described a considerable decrease in the numbers of patients presenting to the Emergency Department with chest pain coinciding with the arrival of COVID-19 and associated lockdown restrictions [2–4]. Scotland is no different with NHS performance indicators reporting that Emergency Departments experienced a substantial reduction in attendances during government advice recommending strict social distancing during the COVID-19 pandemic [5]. This has led to concerns that patients with significant illness such as myocardial infarction may not be attending hospital. National data also reveal excess total mortality that is not completely attributable to COVID-19 [6] suggesting additional public harm may be resulting from decreasing Emergency Department attendances for non-COVID associated illness.

Scotland entered lockdown on 23^rd^ March 2020 with advice to ‘stay home, protect our NHS, save lives’. Patients admitted to acute medical units in NHS Lothian, Scotland, during the first 31 days after lockdown were of higher medical acuity and had a higher risk of inpatient mortality when compared to patients in the same period in the preceding 5 years [7]. This suggests that patients may not have been seeking and accessing healthcare in the same way as prior to the COVID-19 outbreak. It is imperative that patients with chest pain seek professional healthcare assessment for what could potentially be a medical emergency which, if not treated, can lead to life threatening complications such as heart failure, arrhythmia and death.

Previous research on decision making in response to chest pain has revealed a complex series of actions. Patients perform a process of symptom interpretation and self-evaluation of coronary candidacy to assess personal risk [8], they often engage in lay consultation [9], followed by an evaluation of the appropriate course of action based on a personal justification to seek and use healthcare services [10]. Additionally, the context of the acute event also impacts on how the individual responds [11].

**Fig 1:**
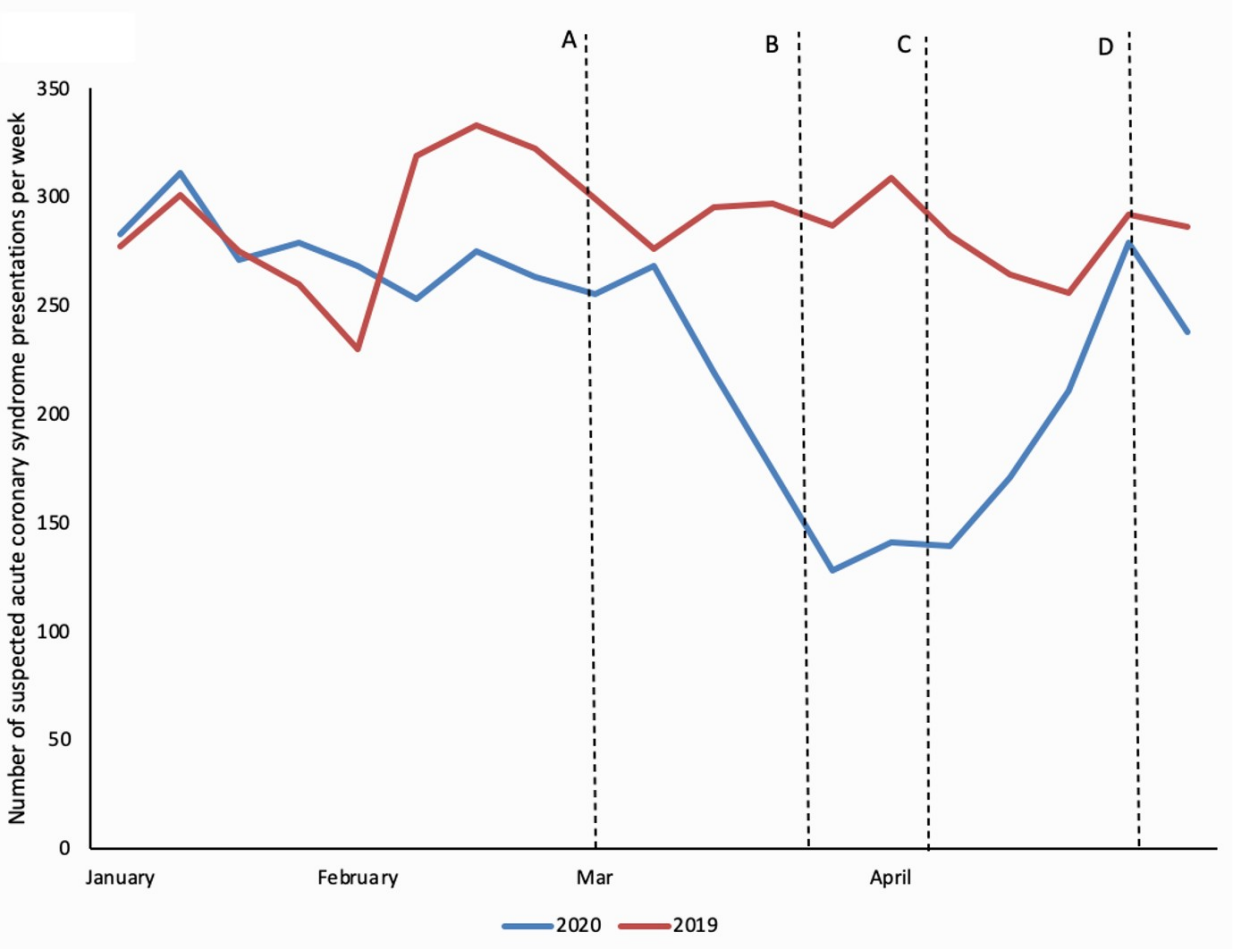
Emergency Department presentations to NHS Lothian per week with suspected acute coronary syndrome from January to May 2019 and 2020. Key dates are denoted by ‘A’ first confirmed COVID-19 case in Scotland 02 March 2020 [12], ‘B’ lockdown enforcement 23 March 2020 [13], ‘C’ British Heart Foundation and media reports of decreased hospital presentation 03 April 2020 [14], ‘D’ ‘The NHS is open’ campaign launched 26 April 2020 [15].

Internal audit data from our own centre revealed the average weekly number of Emergency Department attendances with suspected acute coronary syndrome fell from 287/week between January and May 2019 to 233/week in 2020. The lowest number of attendances per week (128) was seen in the last week of March 2020 as lockdown was announced (unpublished data) (Fig 1). Google trends data confirmed that online searches for the term ‘chest pain’ in Scotland increased during March and April 2020 confirming that the prevalence of chest pain in the community had not decreased so could not explain the decrease in presentations.

These data highlighted the need to explore how and why the COVID-19 pandemic and public health advice had affected chest pain presentations and help-seeking behaviour at an individual patient level using a qualitative interview approach.

## Methods

Single semi-structured telephone interviews were conducted with patients attending hospital for the assessment of suspected acute coronary syndrome between the 17 April 2020 and 08 May 2020. Participants were identified using an order request system for cardiac troponin which identifies all patients with suspected acute coronary syndrome in our centre and permits review of the electronic patient record [16]. Convenience sampling identified a sample of 21 participants. A further 7 telephone interviews were conducted with community-based patients with known coronary heart disease. The rationale for including these patients was to capture a sample of patients who may have experienced chest pain but who chose not to attend hospital during this period. Participants were contacted by telephone and the purpose of the call explained. They were informed that participation was entirely voluntary, and, if happy to participate were asked if they consented to the conversation being audio recorded. All those contacted were happy to participate and for the conversation to be audio recorded. This consent was deemed proportionate to the study activity of a short telephone conversation by the local Quality Improvement Team. A topic guide (S1 Appendix) was developed by the study team comprising of two consultant cardiologists and two nurses and discussed with a patient group. All interviews were conducted by AVF, a female cardiology research nurse with extensive experience in qualitative interviews. Participants were telephoned at home. It is unknown whether anyone else was present with the participant at the time of interview. Interviews lasted 5 to 37 minutes (mean 12 minutes). The interviewer was unknown to participants and was introduced as a cardiology research nurse with an interest in patient response to chest pain. In addition to the conversation being audio recorded, notes were made during the interview. Recruitment continued until saturation was achieved and additional interviews did not yield new insights [17]. Participants were asked to talk about how they made the decision to attend hospital for assessment and whether coronavirus had impacted that decision making process. Participants identified from the community were asked if they had experienced symptoms in the preceding two months and whether coronavirus had impacted how they dealt with those symptoms.

This project was reviewed by NHS Lothian Research and Development Office and the South East Scotland Research Ethics Service. These bodies advised the project was service evaluation therefore research ethics committee approval was not required. It was registered with and approved by the local cardiology Quality Improvement Team according to local practice.

### Data analysis

This study was guided by the principles of phenomenology [18] aiming to explore the form of an event or experience. Analysis was interpretivist and abductive in nature seeking to identify meaning from accounts with reference to prior knowledge from the field [19]. Detailed notes were written with salient issues noted whilst listening to the whole voice files. Notes were read repeatedly searching for patterns of meaning in the data. Data were coded by AVF and codes grouped to identify themes derived from the data. Verbatim extracts were documented as necessary to illustrate themes arising [20]. Summary sheets of interviews were created and emerging themes were discussed with FS and MAD. These discussions also served to promote researcher reflexivity. The emerging themes were not discussed with participants.

### Patient and public involvement

The project was conceptualised through discussion with patients admitted to a cardiology ward during the COVID-19 pandemic. Key ideas for interview questions were developed by consultation with the patient group.

## Results

### Characteristics of interview participants

Interview participants were aged between 39 and 88 years and 54% were female. 14 participants had an admission troponin greater than the diagnostic threshold for myocardial infarction, 7 had an admission troponin less than the diagnostic threshold for myocardial infarction and 7 were under the care of a cardiologist as an outpatient.

### Semi-structured interview results

**Fig 2:**
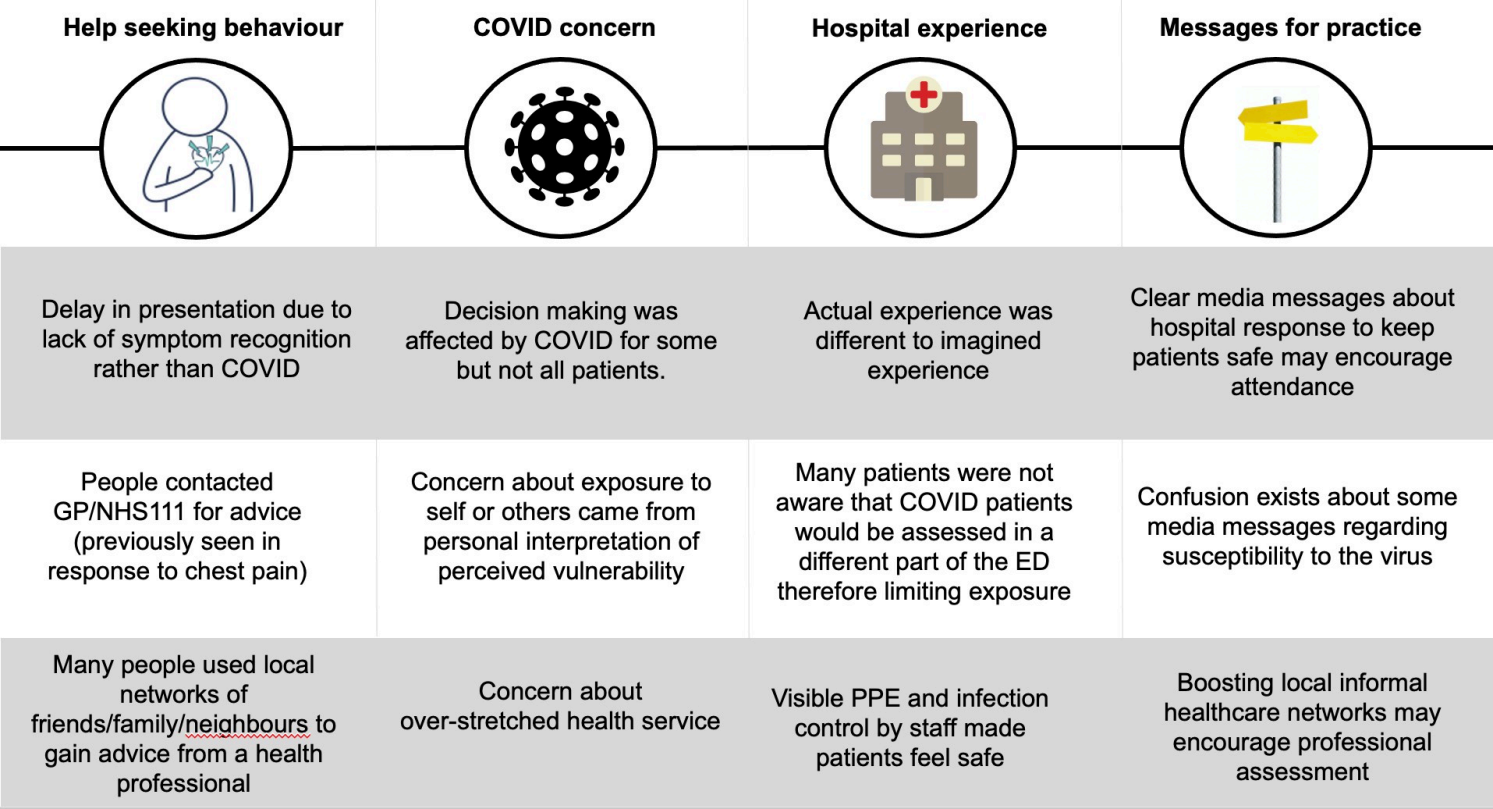
Summary of main interview themes and lessons for practice.

Telephone conversations revealed three main themes; pandemic shaping of help seeking behaviour, COVID exposure concern, and hospital experience. These are summarised in Fig 2 with possible implications for practice.

#### Main theme: Pandemic shaping of help seeking behaviour

This theme describes how participants made the decision to seek professional healthcare assessment. It is divided into subthemes describing a staged response to help seeking; 1) symptom appraisal, 2) consultation of lay members for advice, and 3) accessing professional healthcare assessment. Quotations illustrating these interpretations are given in **Table 1**.

**Table 1:** Theme: Pandemic shaping of help seeking behaviour.

Theme: Pandemic shaping of help seeking behaviour		
Subtheme	*Illustrative quote*	Participant number (sex, age) diagnosis
**Symptom appraisal**	*I’d had a kind of heartburn two days before*. *Then when I was walking the dog I’d got this pain in my chest again*. *I didn’t know what to do due to coronavirus*. *I had pain in my arm the next morning so I phoned the GP*.	2 (M, 59) myocardial infarction
	*The pain wasn’t severe*. *It was coming and going*. *It was what I would call a minor chest pain*.	4 (F, 76 atypical chest pain
**Lay consultation**	*I’d been out in the garden and came over feeling really sweaty and unwell*. *My wife said I looked pure white*. *Our neighbour is a GP so my wife phoned her and asked her what to do*. *She said to phone 999*.	12 (M, 55) myocardial infarction
	*I knew it was a heart attack as I had one four years ago*. *I was thinking ‘why is this happening at this time*?*’ My daughter is a nurse so I told her*. *She gave me aspirin and phoned the ambulance*.	7 (F, 59) myocardial infarction
**Accessing professional assessment**	*They took my BP and my oxygen but didn’t do a trace of my heart*. *They were paranoid about COVID*.	8 (F, 39) myocardial infarction
	*The only thing he did was put a thing on my finger and take my temperature*.	20 (F, 82) Atrial fibrillation

#### Subtheme: Symptom appraisal

Firstly, participants reported performing a symptom appraisal which could lead to symptoms being attributed to other causes, for example indigestion, due to the transient nature of their symptoms or lack of severity. Some participants had experienced myocardial infarction previously and performed a comparison between prior experience and their current symptoms. Based on their self assessment of symptoms, participants then progressed to the next stage.

#### Subtheme: Lay consultation and access to assessment

Persistence of symptoms triggered discussion with local neighbourhood networks and family to decide on the next course of action. It was noted that for the participants in this study, these networks often included a healthcare professional who happened to live closeby. The subsequent outcome of this discussion typically involved contacting a GP or the NHS out of hours service (NHS24) for preliminary assessment. No barriers to accessing these services were reported. Very few participants telephoned the Scottish Ambulance Service directly without additional assessment, with only a few self-presenting to a hospital Emergency Department.

#### Subtheme: Accessing professional assessment

Some participants reported that gaining access to physical examination by a healthcare professional was limited. Consultations tended to be telephone based which were sometimes viewed as inadequate due to difficulty in describing symptoms over the telephone to a doctor with whom they did not have a relationship. Other participants did have access to face-to-face primary care appointments but similarly these were not viewed positively due to lack of physical examination.

One patient (female, 39 yrs) described having three GP phone consultations and an out of hours appointment where she had blood pressure and oxygen saturations recorded but no ECG performed despite describing chest, arm and jaw pain. This patient subsequently self-presented to the Emergency Department with a confirmed acute myocardial infarction.

#### Theme: COVID-19 exposure concern

Participants were asked whether coronavirus, and the societal-response to it, had affected their decision making to attend hospital. Participants differed in their response with some stating their decision making was completely unaffected. Some participants were very concerned about presentation to hospital due to COVID-19, however, it did not stop them attending. Concerns could largely be attributed to three subthemes; perceived vulnerability to the virus, wishing to protect others, and avoidance of adding pressure to busy health services. These will be considered separately with quotations used to illustrate interpretations given in **Table 2**.

**Table 2:** Theme: COVID-19 exposure concern.

Theme: COVID-19 exposure concern		
Subtheme	*Illustrative quote*	Participant number (sex, age) diagnosis
**Perceived vulnerability**	*I never gave it a thought*.	6 (F, 68) heart failure
	*I’m 75*, *I’ve done well to get this far*. *If I get it*, *I get it*.	19 (M, 76) ischaemic heart disease
	*I wouldn’t stop going to hospital because if there was something wrong with me*, *I would certainly go*. *I wouldn’t say I can’t go out because of a virus*.	24 (F, 71) ischaemic heart disease
	*I think I’m more at risk down the street of being exposed to the virus*.	25 (M, 73) ischaemic heart disease
	*I wouldn’t be worried*, *not at all*. *An emergency is an emergency*.	28 (M, 71) unexplained chest pain
	*They are saying the older you are the worse you take coronavirus and I’d been pretty good up until that point at not going into shops*.	4 (F, 76) atypical chest pain
	*Chances are that if I got the virus I would be on a ventilator and at 76 I wouldn’t be a top priority*. *I’m quite realistic on these things*.	9 (F, 76) mitral regurgitation
	*Why is this happening at this time*? *Why do I have to go to hospital at this time with the virus*? *That is what is scaring me the most*.	7 (F, 59) myocardial infarction
	*The one thing that goes through your mind is when you’re having one of those episodes you become more vulnerable no matter what age you are*, *as you have a pre-existing condition then…*..*When you’re having a heart attack your immunity levels go down and the last place you want to go is the biggest place in the city where there’s a massive amount of it [the virus]…Key words stick in your mind…You put yourself in that vulnerable category*.	2 (M, 59) Myocardial infarction
	*Did I want to risk putting myself in the heart of it by going into A+E that night*? *I know how vicious the virus is*.*But*, *if I want to survive*, *I know I have to get myself there*.	9 (F, 76) mitral regurgitation
**Protecting others**	*Fear of compromising someone else is a very real fear*	5 (F, 59) Non-specific chest pain
	*I was prepared not to go because of corona…*.*this was another reason why I didn’t want to go [living with someone shielding]*. *I could have brought the virus into the house and that really concerned me*.	17 (F, 63) unstable angina
**Adding pressure to the health service**	*The number of people with coronavirus and numbers going into hospital*, *and the arrangements the health service were making to cope made you think you shouldn’t put them under pressure*. *They are over exposed*. *At the start it was ‘stay away’*	27 (M, 61) ischaemic heart disease
	*In the beginning I was very concerned*, *and concerned about putting pressure on the GPs as well*, *but I don’t feel so concerned about that now*. *The numbers of people that are in hospital are going down and there has been some publicity about certain areas of the hospital not being too busy and people not going to A&E when they should have been going*.	26 (F, 82) Ischaemic heart disease

#### Subtheme: Perceived vulnerability to the virus

Participants spoke about how they believed their personal vulnerability to the virus and access to treatment would be influenced by their increased age. Some participants believed attending hospital would increase their exposure to coronavirus and discussed the possible repercussions of this with reduced access to ventilators with increased age. One participant (male, 62 years, heart failure) felt at increased risk of contracting COVID-19 after sharing a hospital room with three elderly patients. He cited media reports that elderly people were more at risk of severe COVID-19 disease and interpreted that to mean a greater risk of contracting COVID-19 by sharing a room with elderly people.

Participants also expressed their vulnerability when talking about pre-existing health conditions. One participant (female, 59 years, myocardial infarction) felt vulnerable to COVID-19 due to an impaired immune system and a previous myocardial infarction. A family decision was made to limit her potential exposure to coronavirus from her daughter, a nurse, by leaving the family home to live in temporary accommodation. Whilst this participant did attend hospital for assessment, she stated that she was frightened. Another patient (male, 59 years) who attended hospital with myocardial infarction stated that suffering an acute cardiac event made him more vulnerable to coronavirus and he now considered himself to be in a more vulnerable category.

A further participant (female, 76 years) had two ED chest pain presentations during lockdown. Initially she was assessed and discharged then represented two weeks later with acute myocardial infarction. She knew attending was the most appropriate course of action but described an internal conversation aiming to weigh up the risks of exposing herself to the virus balanced against the risk of not seeking assessment for chest pain.

#### Subtheme: Protecting others from infection

Protecting others was another consideration for participants. One participant (female, 80 years, non-cardiac chest pain) actively wanted to attend hospital to access a test for COVID-19 so she knew she was not putting her carers at risk. Other examples included considering the exposure risk to grandchildren at home, and inadvertently transmitting the virus from the hospital environment to a vulnerable adult in the community by choosing to attend hospital.

#### Subtheme: Adding pressure to the health service

Some participants explicitly stated they were not concerned about adding pressure to health services by attending the ED. They described feeling so unwell they knew they had to attend hospital. Others had learnt that Emergency Departments were quiet through discussions with their GP or local networks which included health professionals. Participants also stated they knew that hospitals were fully open.

For others, media images such as those being reported in Italy were a factor in their reluctance to attend hospital. Daily news reports detailing the number of new cases and deaths, the building of new emergency hospitals with large capacity and images of staff wearing protective suits all contributed to the message of ‘Stay Away’ at the beginning of the pandemic. Participants stated how their perception of this message had changed over time. Publicity about decreased hospital demand was cited as a reason why some participants who would have been reluctant to use services in the beginning were now less concerned about doing so.

#### Theme: Hospital experience

Participants reported a much more positive hospital experience than they had anticipated. They stated the ED assessment areas were quiet and that they were seen quickly. Some were not aware that patients with COVID-19 symptoms entered the hospital and were assessed through a different point of access. Once in hospital they could see they were separated from suspected COVID-19 patients. Some participants were informed by their GP that this would be the case, others said they assumed the NHS would take this action. Quotations used to illustrate these interpretations are given in **Table 3**.

**Table 3:** Theme: Hospital experience.

Theme: Hospital experience	
*Illustrative quote*	Participant number (sex, age) diagnosis
*If anything*, *it wasn’t as busy which meant I was seen a bit quicker than usual*	1 (M, 73) non-cardiac chest pain
*Seeing the TV program in Italy brought home how bad it is*. *It was very different when I got there as everything was segregated off*. *I didn’t know that would happen*. *It wasn’t busy*. *They were all taking precautions*.	20 (F, 82) atrial fibrillation
*Common sense tells you that the NHS wouldn’t be what it is unless they did keep people separate*	23 (M, 71) ischaemic heart disease
*I felt like I was in the safest place in Edinburgh*. *It was very different from what I thought it would be*.	2 (M, 59) myocardial infarction
*It felt safer than usual*. *Hospitals aren’t normally the cleanest places*, *in my opinion*, *so it felt better than usual*. *The cleanliness and what the staff were doing was absolutely spot on*. *You could tell people were taking extra care*	8 (F, 39) Myocardial infarction

Many participants reported feeling safe while in hospital due to regular changing of personal protective equipment and hand washing by staff, in addition to highly visible cleaning taking place.

One participant commented that nurses in the hospital ward were not social distancing. It was also mentioned that not being able to have anyone accompany you to the ED or visit you in hospital made an already worrying time even more difficult.

## Discussion

Key public health strategies were targeted at decreasing community transmission of SARS-CoV-2 included hand washing, social distancing and self-isolation. Mass media campaigns have previously been shown to elicit potentially beneficial behaviour change in response to the SARS and H1N1 epidemics regarding hand washing and social distancing [21]. Governments across the globe used media briefings to keep the public informed of national developments in the fight against SARS-CoV-2 and to promote these key public health interventions. In Scotland, a slogan was developed to reiterate the fundamental message of lockdown; ‘Stay at home to protect our NHS and save lives’. The observed reduction in chest pain presentations to hospital appears to align closely with the dates of public health messaging campaigns (**Fig 1**) suggesting that these may have impacted help seeking behaviour for chest pain during the early stages of the COVID-19 pandemic.

The majority of participants first sought an assessment of chest pain through primary care services. Reluctance to use the emergency services has been seen prior to the COVID-19 pandemic due to concern about appropriate use of the NHS and resources [10, 11]. It appears likely that that these concerns have been enhanced during the COVID-19 pandemic. Parallels can also be drawn with the Three Delays Model of help -seeking [22] which states delays in help-seeking can occur when deciding to seek care, reaching the healthcare facility, and/or receiving care. The participants in this study reported delay in deciding to seek care, but did not report challenges with reaching the healthcare facility or receiving care. Additionally, our findings have highlighted the importance of local neighbourhood networks in providing advice out with a formal healthcare service framework. These networks which consisted of family, friends and neighbours working as doctors, nurses and physiotherapists, were aware of how hospitals were managing admissions through the ED and were often aware of how local services were coping with COVID-19 admissions compared to other hospitals in the UK and around the world. Decision making on whether to attend hospital was therefore typically shifted to an informal health professional network, with current knowledge and understanding of how the healthcare system was functioning during the pandemic. After such informal consultations, participants were often encouraged to attend primary care or the Emergency Department for assessment. Help-seeking is described as involving three distinct elements; the person who is looking for help, the problem for which help is sought and the person from who help is required [23]. Community and social networks have previously been shown to influence help seeking [24]. Interaction with a third party may be facilitated if already known to the person requiring help. It is possible that those who chose to attend the Emergency Department during these unprecedented times were those who were able to access healthcare advice through their own informal networks. Patients with chest pain not attending for assessment may not have had the benefit of an informal local advice structure. This hypothesis could be investigated further by performing an analysis of social deprivation status of participants attending hospital during this time and comparing to the population attending in previous years. We hypothesise that there would be a larger decrease in presentation from areas of higher social deprivation. Additionally, this work included a convenience sample of participants but future work using a purposive sample of participants selecting those from more deprived areas could explore the concept of access to informal healthcare networks further.

As the actual hospital experience was often very different to the imagined experience, and usually positive, it may be useful for future media and government message campaigns to outline clearly a step by step mechanism by which people can access emergency services and to clearly describe safety measures adopted in Emergency Departments to minimise risk for patients that need to attend hospital urgently during future pandemic events. Commercial sectors of society, for example supermarkets, have done this with television campaigns. A ‘Ways we are keeping you safe’ campaign highlighting that hospitals have taken steps to create separate emergency assessment areas into COVID-19-free zones may make patients feel more comfortable attending the hospital.

Patient concerns regarding ‘vulnerability to the virus’ has emerged as an important discourse during the pandemic possibly due to a lack of clarity on which categories of patients were and were not included in government-defined vulnerable groups and the coverage of this topic in the media. For example, a government spokesperson gave confusing messages regarding the inclusion of people over 70 years of age in the vulnerable category [25]. Participants therefore frequently used their own interpretation of government messages and media articles to categorise themselves as vulnerable which contributed to decision making during their chest pain episode. The health belief model is one of the most widely used models for understanding health behaviours often used in the disease prevention and health promotion spheres [26]. The model defines key factors that influence health behaviours which include an individual’s perceived threat to disease (perceived susceptibility), and belief of consequence of that disease (perceived severity). If a person perceives a threat to their health, and their perceived benefits outweigh the perceived barriers (receiving assessment for acute coronary syndrome versus the exposure to Sars-Cov-2) then they are likely to undertake the recommended preventive health action, attending a hospital ED for example. Some participants were therefore faced with having to weigh up the consequences of not seeking assessment for chest pain with potential exposure to SARS-CoV-2. While pandemic communication strategies and policies are selected based on the potential of a positive effect e.g. the stay at home message aiming to decrease the chance of community transmission [27], these same strategies can produce unintended negative effects [28]. Uncertainty regarding personal vulnerability to the virus may have been exacerbated by initial uncertainty as to who was clinically vulnerable due to the unknown nature of the virus [29].

### Limitations

While we aimed to capture the experience of patients who chose not to come to hospital with symptoms of chest pain by targeting those with known coronary heart disease from a community setting, none of the participants in the sample had experienced chest pain during the study period for which they would have normally sought hospital assessment. However, this service evaluation project has revealed valuable insights into how the decision to attend hospital was shaped by the pandemic. The first interviews were carried out at or just after the time of the release of a public health messaging campaign to promote attendance to the ED for urgent conditions. While some interviews included participants who had experienced symptoms two weeks earlier, perceived decision making may have changed in response to evolving media and news campaign. We did not explore factors influencing help seeking behaviour and hospital attendance during the early media campaigns advising patients to stay at home and protect the NHS.

## Conclusion

Future media and public health campaigns associated with subsequent waves of COVID-19 infection should seek to strike a balance between appropriate care-seeking and avoidance behaviour. Such campaigns should be designed to include dynamic monitoring of the public response to healthcare messaging in a way that permits rapid adjustment to ensure that the net impact on health, pandemic and non-pandemic related, is optimised.

## Supporting information

S1 Appendix Interview topic guide. (DOCX)
